# First bacteraemic human infection with *Escherichia albertii*

**DOI:** 10.1016/j.nmni.2015.07.003

**Published:** 2015-07-16

**Authors:** T.J.J. Inglis, A.J. Merritt, N. Bzdyl, S. Lansley, M.N. Urosevic

**Affiliations:** 1) Department of Microbiology, PathWest Laboratory Medicine, Nedlands, Western Australia, Australia; 2) School of Pathology and Laboratory Medicine, Faculty of Medicine, Dentistry and Health Sciences, University of Western Australia, Crawley, Western Australia, Australia

**Keywords:** Bacteraemia, *bacterial identification methods*, *Escherichia*, facultative anaerobes, gut microbiota

## Abstract

The facultative anaerobic Gram-negative species *Escherichia albertii* has been isolated from human faeces in gastrointestinal infection and from a range of wild bird species. Here we report the first case of a febrile infection associated with *E. albertii* bacteraemia in a 76-year-old woman with gastric dysplasia.

*Escherichia coli* is the single commonest Gram-negative species isolated from bacteraemic patients in most hospital centres. Other *Escherichia* species are much less common and are possibly overlooked unless detailed identification procedures are carried out in all cases of presumptive *Escherichia* species bacteraemia. *Escherichia albertii* was first described in 2003 in association with gastrointestinal infection in Bangladeshi children [1]. The species was subsequently identified in fatal infections of wild birds in several continents [2] but has yet to be identified in a bacteraemic human infection.

We describe a case of *E. albertii* infection in a 76-year-old woman. The patient had multiple comorbidities: a recent pelvic fracture from which she was convalescing, a dysplastic polyp of the gastric fundus with carcinoma-in-situ, hypothyroidism due to a previous thyroidectomy for papillary carcinoma, epilepsy and hypertension. She was admitted from residential care with a febrile illness of undetermined cause, an oral temperature peaking at 38.7°C, tachycardia at 139 beats per minute and a respiratory rate of 26 breaths per minute. Blood cultures collected during febrile episodes over a 24-hour period resulted in isolation of an oxidase-negative, Gram-negative bacillus from two sets of aerobic and anaerobic bottles inoculated on separate occasions. MALDI-TOF (matrix-assisted laser desorption/ionization time-of-flight mass spectrometry) analysis with direct extraction from blood cultures resulted in presumptive identification of *Escherichia coli* (score > 2.0; Biotyper, Bruker Daltonik, Germany). Targeted identification of blood culture isolates identified *E. coli* (score > 2.0, no alternative species listed). The patient was treated initially with piperacillin/tazobactam intravenously for 72 hours and then after defervescence with oral ciprofloxacin 500 mg twice daily. There were no clinical features of gastrointestinal infection. A midstream urine specimen contained >100 × 10^6^ leucocytes per litre and a few bacteria on centrifuged deposit, but no significant bacterial growth. She defervesced rapidly and made an uneventful clinical recovery.

The possibility of an alternative aetiology was only considered when a molecular method of identifying common causes of bacteraemia (film array; BioFire, bioMérieux, France) produced a discrepant result: positive for *Enterobacteriaceae* but negative for *E. coli.* All isolates were oxidase-negative, Gram-negative bacilli on 5% horse blood agar and initially non-lactose-fermenting colonies on MacConkey agar. We identified them by substrate utilization (API 20E, bioMérieux), resulting in a low discrimination *E. coli* identification (results of tests against were urease positive, rhamnose and melibiose negative). Glucose, lactose and ONPG (O-nitrophenyl-β-d-galactopyranoside) tests in the panels were consistently positive. An isolate extract was prepared for 16S-based identification, which was consistent with *E. albertii* (Fig. 1). Though the patient had no history of gastrointestinal infection symptoms such as diarrhoea, we sought evidence of Shiga toxin [3] by PCR assay and tissue culture cytotoxin assay. Both were negative. The patient had no recent contact with birds, caged or otherwise, young children or other family members with active diarrhoea; no recent history of travel outside the Perth metropolitan area; and no investigations in the preceding months for diarrhoea or other clinical features of gastrointestinal infection. No bird die-off had been reported in the state during the previous 6 months.

The aetiology of this infection would have been missed had it not been for the discrepancy we recorded between the MALDI-TOF results and the film array identification of the blood culture isolates because the urinary leucocytosis was interpreted as presumptive evidence of urosepsis, and the initial identification of *E. coli* isolated from repeated blood cultures was taken as further evidence of urosepsis. The phenotypic features of *E. albertii* are sufficiently variable to render classic phenotypic identification methods unreliable [4], and even the use of substrate utilization leant towards a low-discrimination *E. coli* result for all blood culture isolates. Although the erroneous identification of *E. coli* did not result in inappropriate clinical management, the opportunity for early public health intervention against a cluster of gastrointestinal infection cases might have been missed if the strain had been Shiga toxin positive [5], [6]. It is of particular note the woman had no epidemiologic connection with sick birds or children, as documented in early reports of *E. albertii* infection [1], [2]. *E. albertii* has recently been recognized as an early speciation event close to the separation of *E. coli* and *Shigella* species and has complex genomic diversity [7]. The addition of clinically significant *E. albertii* strains to culture collections used for identification databases will assist in timely recognition of this emerging pathogen and will help clarify whether this species is responsible for a wider spectrum of human disease than originally thought.

## Conflict of interest

None declared.

## Figures and Tables

**Fig. 1:**
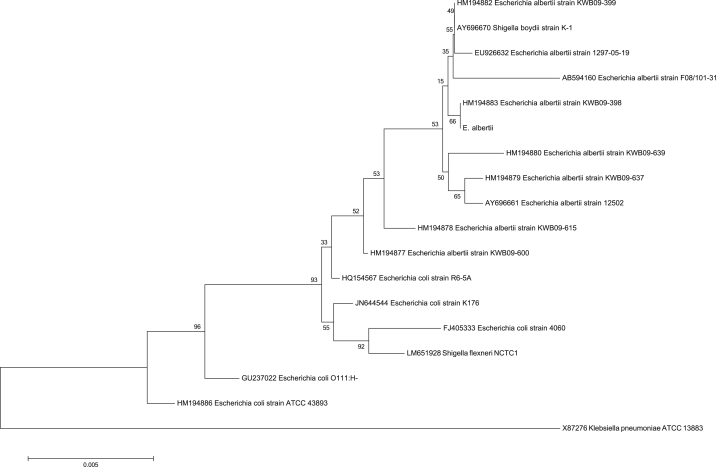
Neighbour-joining tree depicting 16S rRNA gene sequence of STRAIN PERTH and related taxa. GenBank accession numbers are shown in brackets.
